# Compressive and shear behaviour of high-water quick-setting material modified collapsible loess subgrade

**DOI:** 10.1038/s41598-026-42841-0

**Published:** 2026-03-23

**Authors:** Xianwei Tang, Zizhao Zhang, Yuanchang Liu, Kai Chen, Yanyang Zhang, Hongchao Zhao

**Affiliations:** 1 School of Geology and Mining Engineering, Xinjiang University, Urumqi, 830000 China; 2 State Key Laboratory of Intelligent Construction and Healthy Operation and Maintenance of Deep Underground Engineering, Xuzhou, 221116 China

**Keywords:** High-water quick-setting material, Road subgrade, Collapsible loess, Modification technique, Mechanical properties, Economic geology, Structural materials

## Abstract

This paper proposed a conceptual material to construct the road subgrade, for which the high-water quick-setting material is adopted to modify the collapsible loess. To verify the feasibility and easy-to-construct of this innovative construction material, a series of mechanical tests were carried out to investigate the compressive and shear behaviour of high-water quick-setting material modified Ili loess. The results demonstrate that the high-water quick-setting material significantly enhances the early-stage strength of loess. The mechanical analysis indicates that both the unconfined compressive strength and elastic modulus reach their maximum values at a soil-to-binder ratio (S/B) of 3:1, but gradually decrease as the water-to-binder ratio (W/B) increases. Cohesion shows a continuous decline with higher W/B ratio, while the internal friction angle is most significantly improved at the optimal S/B ratio of 3:1. the scanning electron microscope (SEM) results confirmed that ettringite (AFt) and calcium silicate hydrate (C-S-H) gel are key contributors to strength enhancement, with NMR analysis indicating optimized porosity at favorable mix proportions. Water immersion tests identified an optimum formulation that maintained high structural stability under wet conditions, as evidenced by its exceptional softening characteristics. An integrated engineering adaptation model was established by correlating mix parameters with mechanical properties, balancing strength and admixture content. The high-water material demonstrate effective improvement of collapsible loess, significantly enhancing its engineering performance and validating its suitability for loess subgrade construction.

## Introduction

To promote coordinated regional development and meet the needs of constructing the core area of “The Belt and Road”, Xinjiang has continuously strengthened the construction of its transportation network in recent years. By the end of 2023, all prefectural administrative centers in Xinjiang had been connected by expressways, and efforts are now being accelerated to achieve the strategic goal of “Expressways connecting every county”. However, in the Loess collapsible area of Xinjiang’s Ili region, where groundwater levels are high or rainfall is heavy, Loess subgrade is prone to rainwater immersion, leading to Highway subgrade diseases that severely impact Road service performance and Traffic safety. For subgrade diseases caused by loess collapsibility, developing effective methods to quickly treat affected road sections and restore Road structural stability and Service function constitutes a critical Scientific problem urgently requiring resolution in Road engineering maintenance within Collapsible loess area.

Common approaches previously employed for treating soft soil subgrades include physical, chemical, and microbial approaches^1–3^. Physical methods encompass subgrade reinforcement^4,5^, drainage consolidation^6,7^, and vibration compaction^8^. Chemical methods typically utilize materials such as fly ash, lime, or cement^9–11^, with cement and lime frequently selected for improving Loess subgrade in numerous practical projects. However, When employing cementitious materials such as cement and lime to address subgrade collapse issues in loess soils, significant shortcomings exist in curing duration and early strength development, typically requiring 7 to 28 days to achieve design strength^12–14^; Furthermore, these materials necessitate strict control of water content during construction. Excessive moisture occupies pores, retarding cement hydration and reducing the density of the cementitious matrix, ultimately resulting in insufficient strength and compromising treatment efficacy^15^.Therefore, to address the Subgrade diseases caused by loess collapsibility, there is an urgent need for a material with rapid setting time and high early strength.

Existing studies have shown that high-water quick-setting material is a two-component powder material characterized by early initial setting and high strength^16–20^. Through its “active water replenishment + rapid water locking” mechanism, high-water quick-setting material can address the issues of insufficient local solidification and residual porous structures caused by moisture deficiency when loess is using lime and cement. The high-fluidity slurry formed can penetrate micro to macro pores, not only promoting uniform hydration of cementitious components but also enabling the soil to undergo complete wetting and swelling through “pre-water filling”, thereby eliminating potential collapsibility^21,22^. Meanwhile, the slurry can be injected via high-pressure jetting to fill rolling blind areas, forming a soil-slurry interlocking structure that significantly enhances treatment uniformity and compaction while reducing cracking during rapid setting and hydration^23,24^. Additionally, its “rapid setting” property enables mixtures of high-moisture-content materials and soft soil to gain strength quickly. The material typically sets within 3–30 min, begins developing strength within 0.5–1.0 h, and can reach approximately 95% of its 28-day Unconfined compressive strength (UCS) within the first 7 days. Unlike lime or cement used for loess improvement, it avoids slow setting and soft elastic deformation caused by excessive moisture^25^. Furthermore, multiple studies have clarified that the key to the hydration and hardening mechanism of high-water quick-setting materials lies in their rapid hydration to form cementitious products such as ettringite, which enables rapid setting, early strength development, and pore filling^26–28^; preliminary identification has also been made of the W/B ratio and curing conditions that optimize material performance and durability^22–30^.

While current research has established fundamental principles for high-water quick-setting materials, The underlying mechanisms governing material-loess particle interactions and their contribution to macroscopic strength remain unclear. Additionally, the relationship between the W/B ratio, S/B ratio, and mechanical properties requires further investigation. As a solid powder, high-water quick-setting material has the potential to replace cement and lime as a cementitious material for eliminating loess collapse. In the present research, the concept of the innovative modification technique with the application of high-water quick-setting material was proposed to construct the road subgrade. Based on the laboratory investigation in terms of the mechanical properties of the plain Ili loess, the effects of different W/B and S/B ratios under various loading rates was evaluated for these modified loess samples under the uniaxial compression. In parallel, the direct shear tests were carried to analyze changes in the shear strength, cohesion, and internal friction angle. Finally, the SEM analysis was conducted to investigate the influence of the high-water quick-setting material with different W/B ratios and S/B ratios on the microstructure of loess. These findings provide a theoretical basis for understanding the evolution of physical and mechanical properties and the microscopic mechanism of loess modified by high-water quick-setting materials.

## Experimental testing

### Experimental materials

**Fig. 1:**
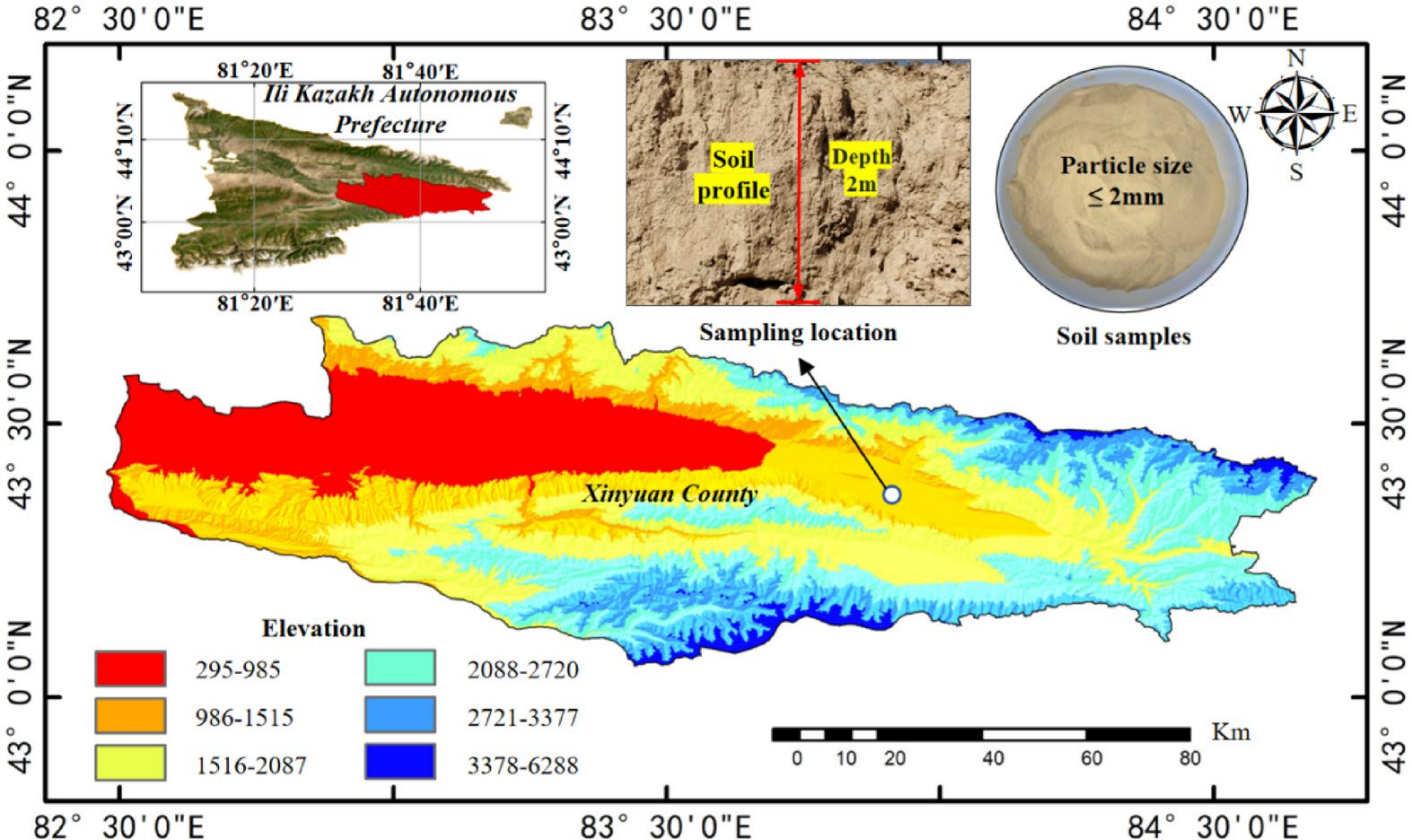
Sampling point location map (It was drawn by ArcMap 10.8. https://www.esri.com).

**Fig. 2:**
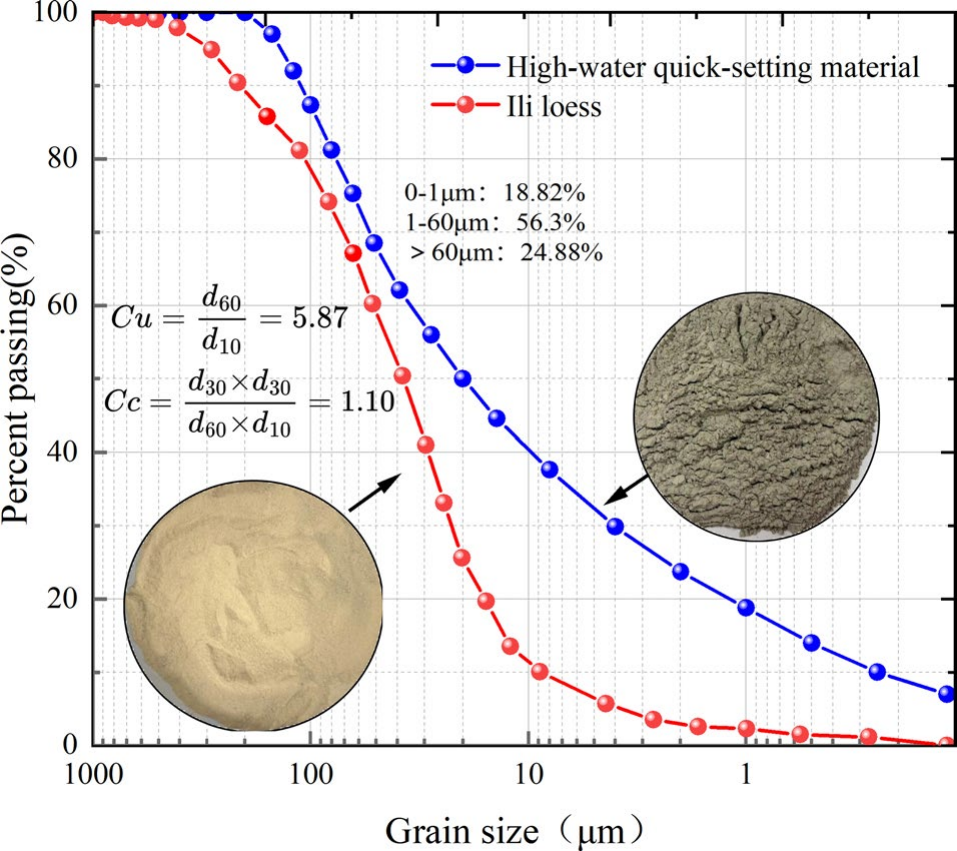
Cumulative particle size distribution curve of the material.

The materials used in the experiments include high-water quick-setting material, Ili loess, and water. The loess samples were collected from the roadside of National Highway 218 in Xinyuan County, Ili region. To minimize potential disturbances from surface-decomposing plants and other environmental factors, the samples were obtained from a 5-m range along the roadside at a depth of 2 m. The loess in this area is pale yellow and has a relatively high natural moisture content, as shown in Fig. 1. The basic physical indexes are measured according to the Standard for Geotechnical Test Methods (GB/T 50123 − 2019). The results of these measurements are provided in Table 1. The dried loess samples were sieved through a 2 mm mesh, and the particle size distribution was analyzed using a laser particle size analyzer. The experimental data were recorded, and average values were calculated, as shown in Fig. 2.

The high-water quick-setting material used in this study was independently developed by the China University of Mining and Technology. It consists of two components, labeled A and B. Component A is primarily composed of bauxite, gypsum, and other independently fired materials combined with a super-retarded dispersant. Component B contains gypsum, lime, and a composite rapid-setting early-strength agent, along with a suspending dispersant. The components are mixed in a 1:1 ratio, and the particle size distribution is illustrated in Fig. 2. X-ray fluorescence (XRF) analysis was performed on the high-water quick-setting material, with the results shown in Table 2.

**Table 1:** Basic physical properties of loess.

Field moisture content	Field density	Field dry density	Saturated water content	Plastic limitw_*p*_	Liquid limitw_l_	Maximum dry density	Optimum moisture content
20.89%	1.96 g/cm^3^	1.64 g/cm^3^	24.57%	17.34%	27.09%	1.86 g/cm^3^	17.40%

**Table 2:** Major chemical composition of high-water quick-setting material.

Main component	CaO	Al_2_O_3_	SiO_2_	Rh	MgO	Fe_2_O_3_	Na_2_O	K_2_O
Percentage%	30.95	16.44	11.76	5.72	3.09	1.48	0.78	0.22

Note: The total sum is less than 100% primarily due to the presence of crystalline water (H₂O) and other light elements not detected by XRF.

### Sample preparation

In accordance with the requirements of geotechnical test standards, on the basis of the determination of basic physical indicators, to mitigate the masking of genuine improvement effects caused by fluctuations in moisture content, loess samples were prepared with the optimum moisture content as the initial moisture content.

**Fig. 3:**
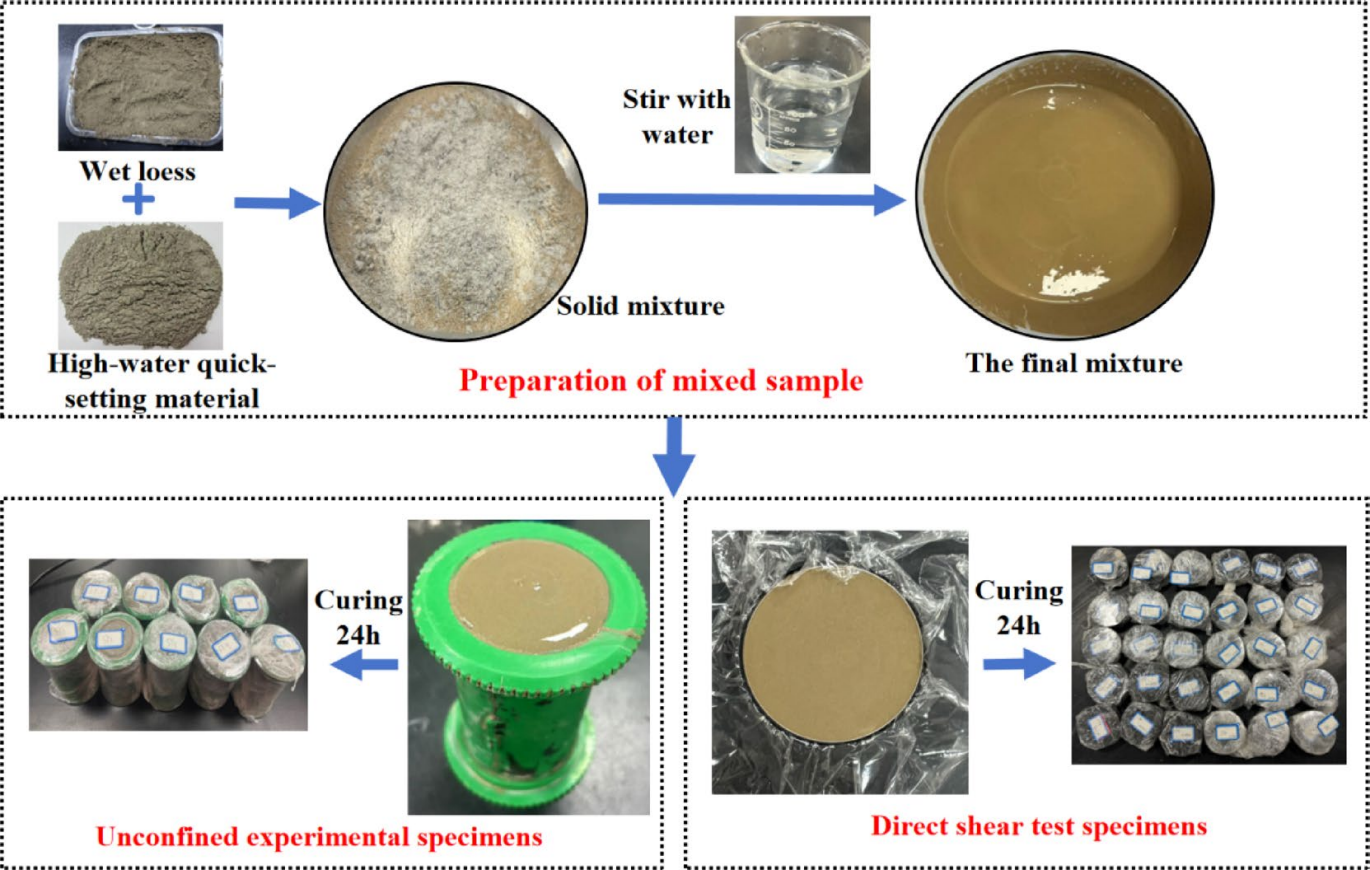
Sample preparation.

Detailed procedures are illustrated in Fig. 3, with specific steps as follows: (a) Loess with a moisture content of 17.4% was prepared and sealed for storage; (b) According to the size of the W/B ratio and S/B ratio required in the experimental design, the weighed loess and the high-water quick-setting material were poured into a basin and stirred thoroughly for 5 min to ensure homogeneous mixing. Subsequently, a predetermined amount of water was added and mixing continued for 5 min. (c) The mixture was divided into three equal portions. Each portion was sequentially poured into a mold. After each addition, the mixture was compacted by vibrating the mold vertically 40 times. Once fully loaded, the upper and lower surfaces of the sample were leveled using a scraper, and the sample was wrapped with plastic film for preservation; (d) All specimens were cured at room temperature for 24 h.

### Experimental apparatus

**Fig. 4:**
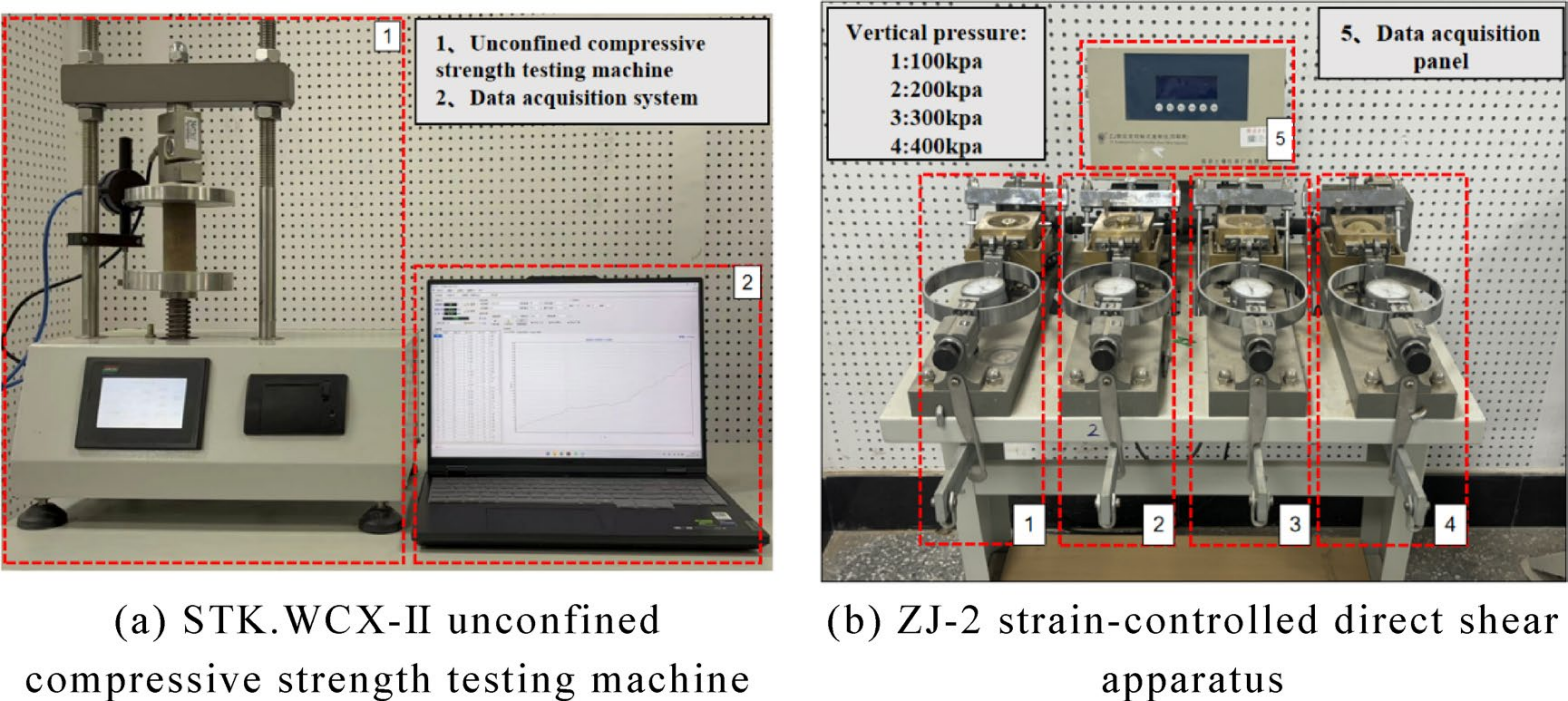
Main experimental instruments.

In this study, unconfined compressive strength (UCS) tests were conducted using the STK.WCX-Ⅱ unconfined compressive strength testing machine, illustrated in Fig. 4a. The equipment includes a pressure application system, lifting mechanism, displacement gauge, and an automatic data acquisition system. It offers a pressure application precision of 0.3% and a displacement accuracy of 0.01 mm. The machine features automatic loading and unloading and is integrated with an automated data collection system connected via STECLAB software, which allows for the setting of experimental parameters and the control of the loading rate. The UCS test specimens were cylindrical, with a diameter of 39.1 mm and a height of 80 mm. A ZJ-2 strain-controlled direct shear apparatus was used for the direct shear test. The apparatus comprises a pushing base, shear box, force measurement ring, lever pressure system, and loading/unloading components. The direct shear test specimens were cylindrical, with a diameter of 69.1 mm and a height of 20 mm. A photo of the direct shear apparatus is presented in Fig. 4b.

### Experimental design

Unconfined compressive strength tests and direct shear tests were conducted in accordance with Standard Test Methods for Soils in Highway Engineering (JTG 3430-2020). According to the findings of zou et al.^31,32^, high-water quick-setting materials exhibit a broad W/B ratio range, which can be adjusted arbitrarily between 0.3 and 3. Prior to the formal experiments, preliminary tests were performed. The results indicated that when the W/B ratio was less than 2:1, a S/B ratio below 6:1 significantly enhanced the unconfined compressive strength of loess, with all specimens achieving final setting within 24 h of water mixing. Consequently, three gradient levels of W/B ratios and S/B ratios were designed based on the preliminary test results. For each group of mix proportion tests, three parallel experiments were conducted to minimize experimental errors.And all specimens were tested at the 24-h age.

1. Unconfined compressive strength test.

As shown in Table 3, an orthogonal experiment with three factors and three levels was designed for the unconfined test, where the three factors are W/B ratio (A), S/B ratio (B), and loading rate (C). A low W/B ratio is used to verify the hydration reaction efficiency and hardened strength of the material under low moisture conditions, while a high W/B ratio simulates its stability and setting performance in water-rich environments. The three S/B ratios help determine the optimal dosage range, ensuring improvement effectiveness while controlling costs. The loading rates are set at 0.5, 1.0, and 2.0 mm/min to simulate the actual strain rate range (10⁻⁴~10⁻²/s) of highway subgrades under vehicle dynamic loads and analyze the material’s mechanical properties and failure mechanisms.

(2) Direct shear test.

**Fig. 5:**
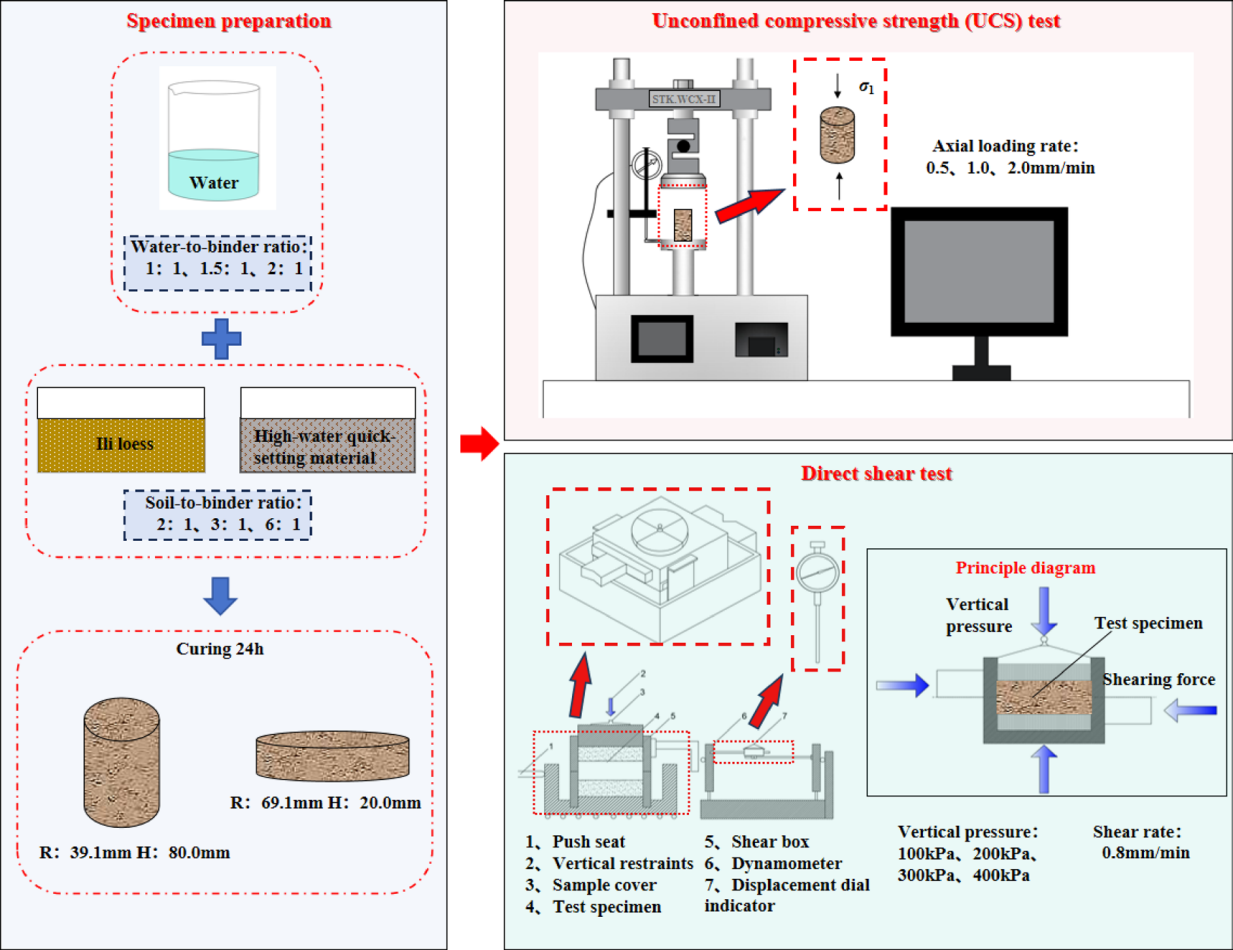
Experimental methodology.

Direct shear tests are used to verify the change in shear strength of 9 sets of samples with the same S/B ratio and W/B ratio as the unconfined compression tests. Therefore, a shear rate of 0.8 mm/min was applied, with shear displacement monitored via dial gauge readings. The test was terminated when the horizontal load stabilized or shear deformation exhibited abrupt acceleration, indicating specimen failure. The experimental framework is summarized in Fig. 5.

**Table 3:** Factor level of orthogonal experimental design.

Sample group number	Factor level		
	W/B ratio (A)	S/B ratio (B)	Loading rate (C) Mm/min
A_1_B_1_C_1_	1: 1	1: 1	0.5
A_1_B_2_C_2_	1: 1	2: 1	1.0
A_1_B_3_C_3_	1: 1	3: 1	2.0
A_2_B_1_C_2_	2: 1	1: 1	3.0
A_2_B_2_C_3_	2: 1	2: 1	2.0
A_2_B_3_C_1_	2: 1	3: 1	1.0
A_3_B_1_C_3_	3: 1	1: 1	2.0
A_3_B_2_C_1_	3: 1	2: 1	1.0
A_3_B_3_C_2_	3: 1	3: 1	3.0

## Results analysis

### Compressive behaviour of high-water quick-setting material modified loess

#### Failure mode

**Fig. 6:**
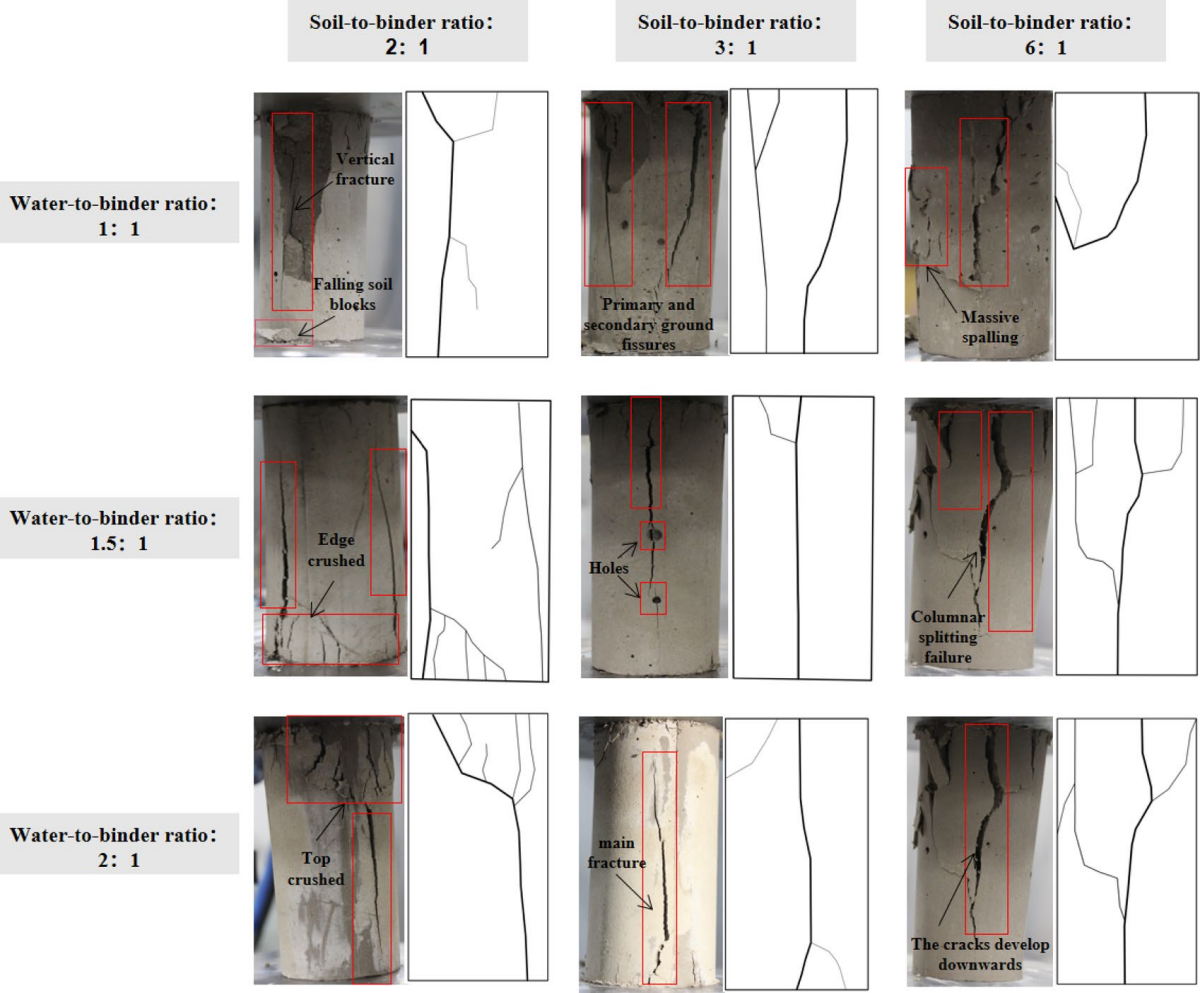
Sample failure modes.

The failure process of high-water quick-setting material-modified loess under vertical loading rate (0.5 mm/min) exhibits distinct stage-dependent characteristics (Fig. 6). Initially, a compaction stage dominates, where the rapidly formed cementation network effectively resists deformation through its skeletal structure. Subsequently, the material transitions to an elastic deformation stage, where the binding network restricts lateral expansion but induces localized microcracks. With increasing load, brittle failure characteristics emerge, marked by vertically penetrating primary cracks along the maximum shear stress plane. Ultimately, the specimen fails via a classic columnar splitting pattern.

This characteristic of ‘initial high strength followed by later brittle failure’ stems from the strong C-S-H gel produced by cement hydration, which provides high bonding strength but increases brittleness. Once a fracture surface forms, rapid failure ensues^33,34^. While this structure provides high early-stage strength, it concurrently reduces material ductility, leading to stress concentration and sudden catastrophic failure under critical loading conditions^35^.

#### Compressive strength analysis

**Fig. 7:**
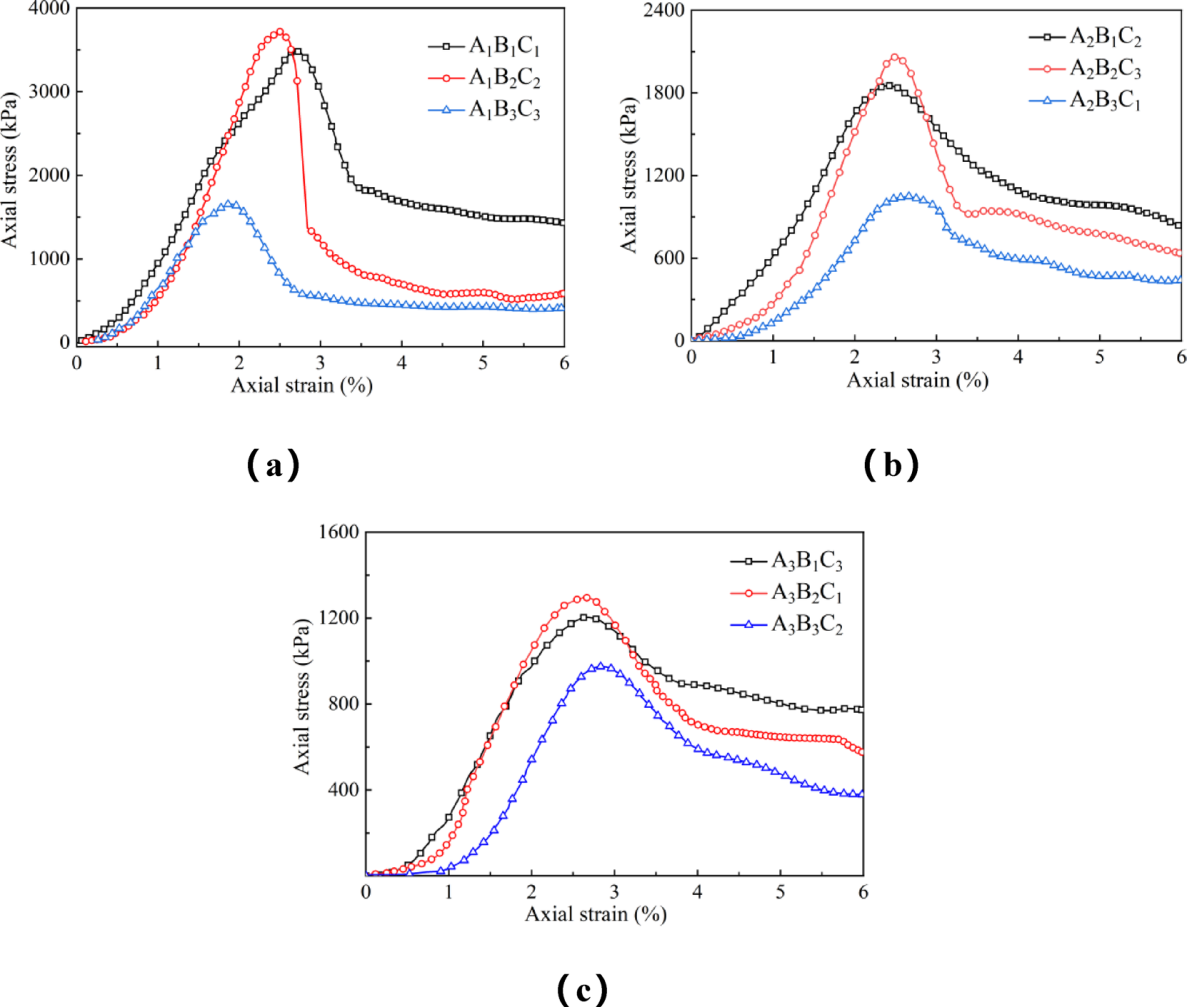
Stress-strain curves.

An analysis of the stress-strain curves for each sample under different experimental conditions is presented in Fig. 7. The unconfined compressive strength tests revealed that all loess samples mixed with high-water quick-setting material displayed a clear peak in the stress-strain curve under unconfined conditions, which is consistent with previous studies^36–38^.

Failure occurred at relatively low strain values (ε < 3%). The stress-strain curves can be divided into four stages. Initially, during the loading and compaction stage, the curve is concave or nearly linear. This stage corresponds to the initial contact between the upper loading plate and the sample, during which the sample is gradually compacted. As the load is applied, the internal voids of the sample are compressed, causing the pore size and the overall volume of the soil to decrease. The next stage is elastic deformation, where the curve becomes linear, and strain increases continuously.

**Fig. 8:**
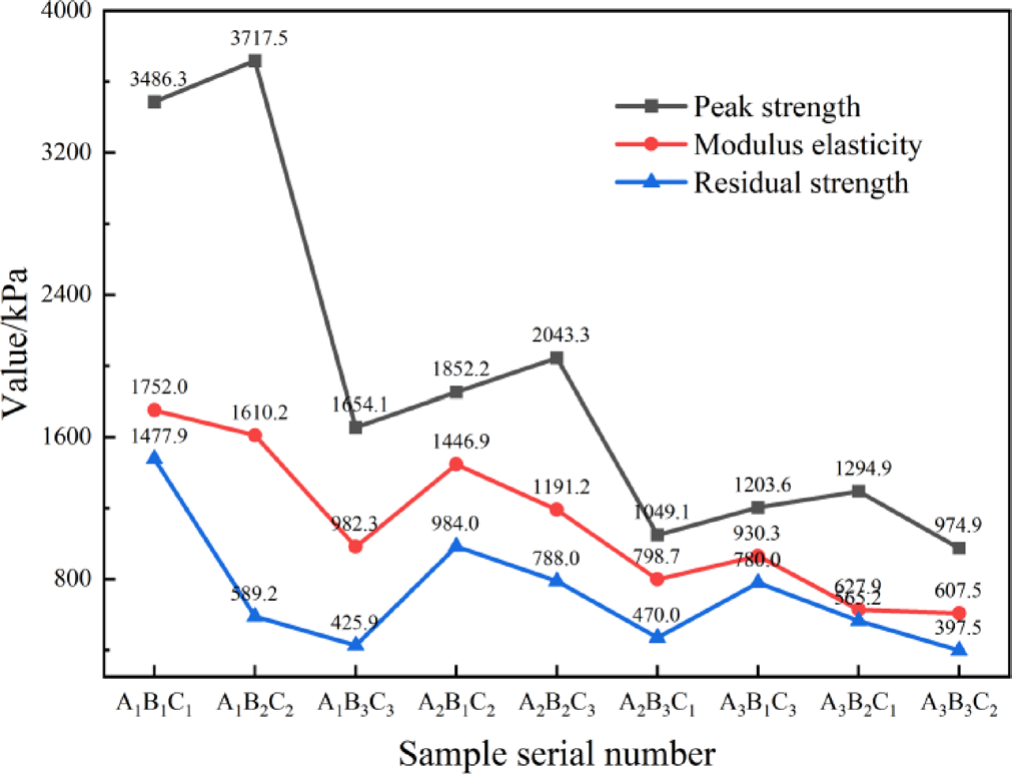
Changes in mechanical properties.

Unconfined compressive strength (UCS) parameters of loess under varying conditions, derived from stress-strain curve analysis (Fig. 7), are summarized in Fig. 8. The elastic modulus was determined from the cut-line modulus of the stress-strain curve. The mechanical properties of the material are significantly influenced by the W/B ratios and S/B ratio^39^. As the W/B ratio increases to 3:1, both the elastic modulus and peak strength generally decrease. Similarly, a higher S/B ratio leads to reduced strength. The brittle index tends to increase at higher S/B ratios, indicating greater susceptibility to brittle failure.The residual strength is calculated according to the minimum stress value when the stress-strain curve tends to be stable after the material experiences the peak strength. The results show that: All specimens showed post-failure residual strength, with A_1_B_1_C_1_ having the highest value. This indicates that modified loess maintains load-bearing capacity after failure. Residual strength decreased as W/B and S/B ratios increased .

#### Sensitivity analysis

##### Range analysis

**Fig. 9:**
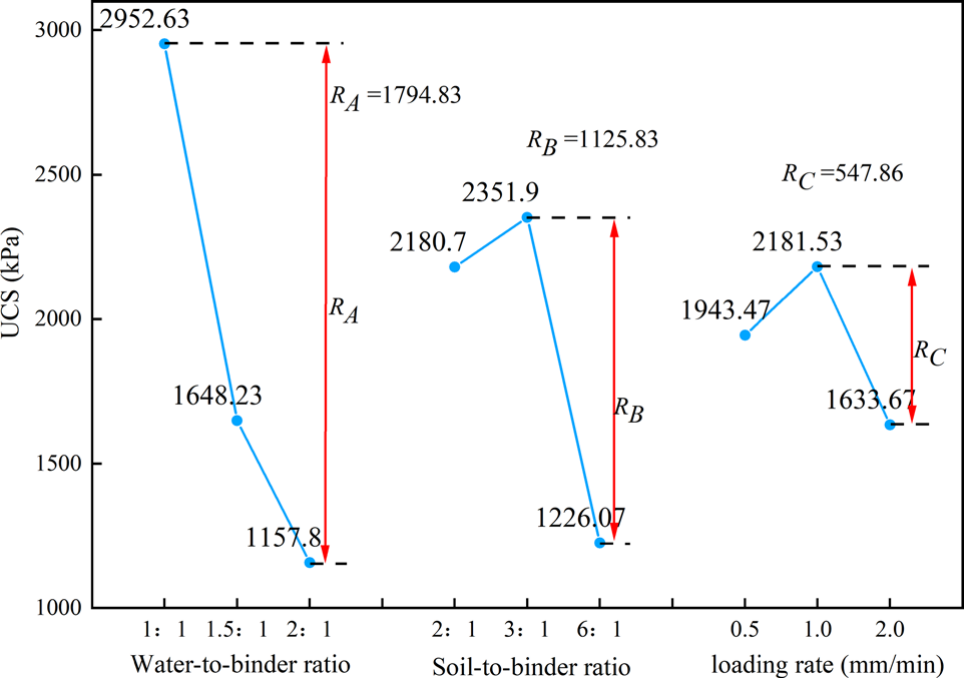
Range analysis of unconfined compressive strength.

As shown in Fig. 9; Table 4, range analysis was employed to rank their sensitivity and evaluate optimal mix ratios, aiming at quantifying the relative influence of key factors on compressive strength. As indicated by the range analysis principle, a larger R value (range) corresponds to a greater overall impact on experimental outcomes—specifically, unconfined compressive strength (UCS). The factors were prioritized in the following order: W/B ratio > S/B ratio > loading rate.

**Table 4:** Range analysis table of unconfined compressive strength.

Item	Level	W/B ratio	S/B ratio	Loading rate
K value	1	8857.9	6542.1	5830.4
	2	4944.7	7055.7	4901
	3	3473.4	3678.2	6544.6
K avg value	1	2952.63	2180.7	1943.47
	2	1648.23	2351.9	1633.67
	3	1157.8	1226.07	2181.53
Bes level		1	2	3
R		1794.83	1125.83	547.86
Number of levels		3	3	3
Repeats per level r		3	3	3

Comparison of mean UCS values across factor levels revealed the optimal design combination as A_1_B_2_C_2_, corresponding to a W/B ratio of 1.0, S/B ratio of 3.0, and loading rate of 1.0 mm/min. Under these conditions, the high-water quick-setting material demonstrated maximal efficacy in enhancing loess compressive strength.

In range analysis, a larger R-value indicates a greater impact on the entire experiment, meaning a stronger effect on unconfined compressive strength^40^. The ranking of the factors based on their influence on compressive strength is as follows: W/B ratio > S/B ratio > loading rate. Comparing the average values at different levels of each factor reveals that the optimal design combination—based on unconfined compressive strength as the evaluation index—is A_1_B_2_C_2_. This combination corresponds to the best enhancement of loess compressive strength when the W/B ratio is 1.0, the S/B ratio is 3.0, and the loading rate is 1.0 mm/min.

##### Analysis of variance

**Fig. 10:**
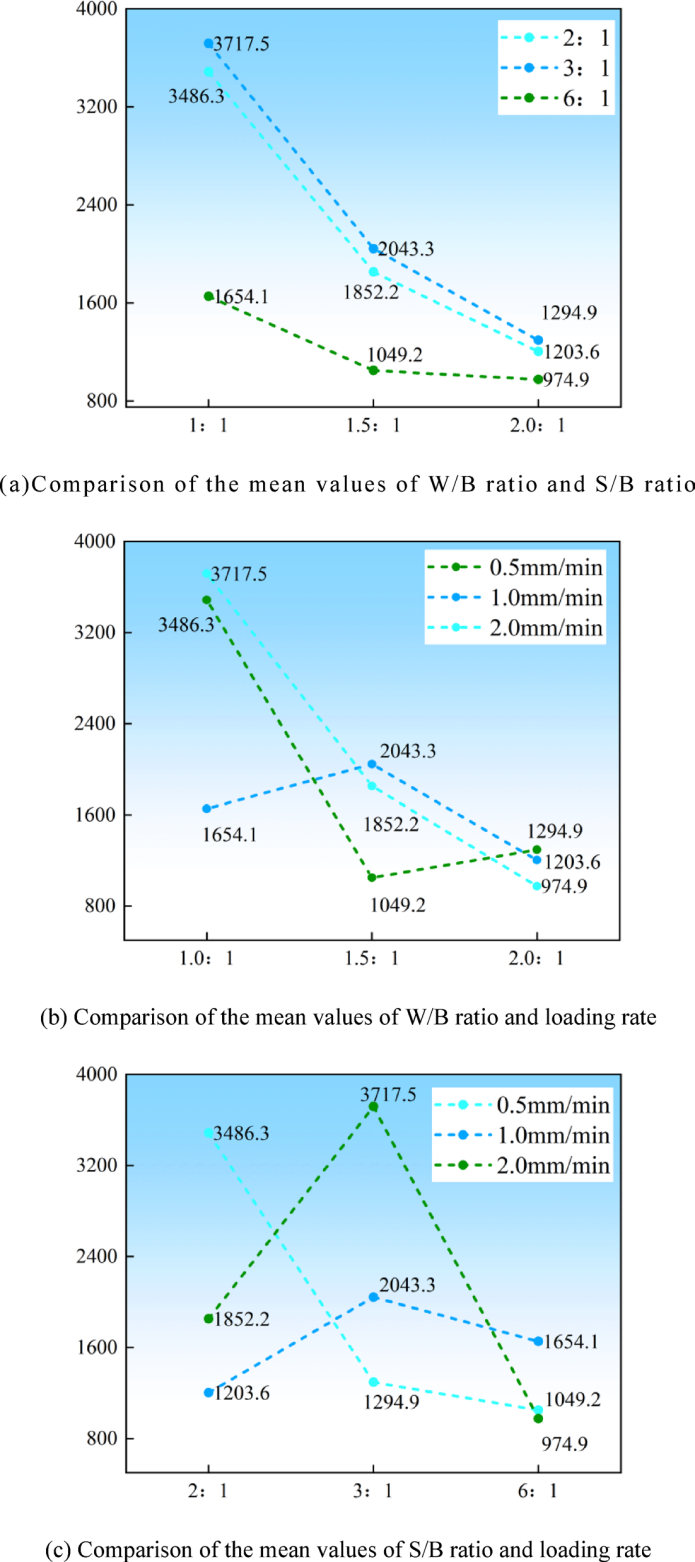
Comparison of mean values of each factor.

While range analysis provides an intuitive and quantitative determination of the primary effects of factors on physical and mechanical properties, it may have some errors and cannot precisely estimate the influence of each factor^41,42^. Variance analysis helps to address this limitation by conducting significance tests^43^. Based on the unconfined compressive strength peak results in Fig. 3, a variance analysis was performed, as shown in Table 5. As can be seen from Fig. 10; Table 5, the three-factor variance analysis (W/B ratio, S/B ratio, and loading rate) reveals the following: The W/B ratio did not show significant effects (F = 10.206, *p* = 0.089 > 0.05), indicating no significant variation in unconfined compressive strength. The S/B ratio also showed no significant effect (F = 4.364, *p* = 0.186 > 0.05), indicating no significant impact on unconfined compressive strength. The loading rate was not significant either (F = 0.895, *p* = 0.528 > 0.05), meaning it does not significantly affect unconfined compressive strength. The F ratio is indicative of the level of influence each factor has on the experiment, with higher F ratios corresponding to greater impact. Therefore, the factors can be ranked based on their F ratios.

**Table 5:** Variance analysis results for unconfined compressive strength.

Source of variation	Sum of squares	df	Mean square	F	*p*
Intercept	33,162,241.78	1	33,162,241.78	131.093	0.008**
W/B ratio	5163,410.909	2	2581,705.454	10.206	0.089
S/B ratio	2208,134.936	2	1104,067.468	4.364	0.186
Loading rate	452,809.662	2	226,404.831	0.895	0.528
Residual	505,932.816	2	252,966.408		

Note: *R*^2^ = 0.939.

*p < 0.05; **p < 0.01.

It is obvious in Table 5, the ranking of factors affecting unconfined compressive strength is as follows: W/B ratio > S/B ratio > loading rate. This result is consistent with the range analysis findings in Table 4. Based on this analysis, the optimal mix for achieving maximum unconfined compressive strength is determined by selecting the factor levels that correspond to the highest compressive strength: a W/B ratio of 1.0, a S/B ratio of 3.0, and a loading rate of 1.0 mm/min.

### Shear behaviour of high-water quick-setting material modified loess

#### General observation

**Fig. 11:**
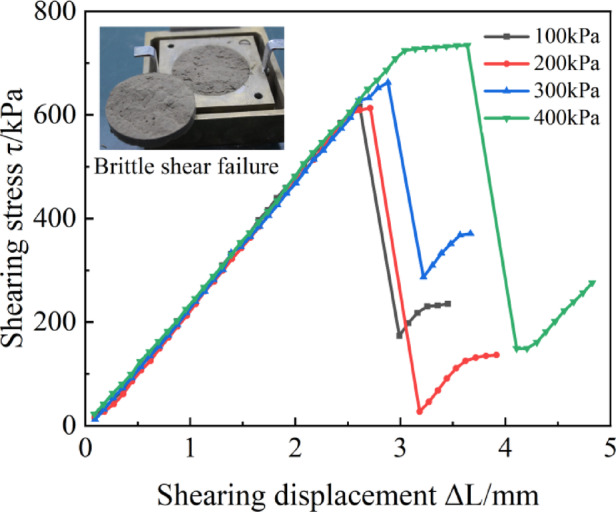
Typical stress-strain behaviour.

Direct shear tests demonstrated that all specimens exhibiting typical brittle shear failure. Representative stress-displacement curves (Fig. 11) revealed a marked increase in shear strength with rising normal pressure. Within the tested normal pressure range (100–400 kPa), strength growth followed the Mohr-Coulomb strength theory, indicating enhanced stability of the cementation network under elevated pressures.The curves displayed sharp stress peaks followed by abrupt post-peak softening, with failure displacements confined to 2–4 mm. This rapid stress drop signifies sudden fracture of the cementation network.

**Fig. 12:**
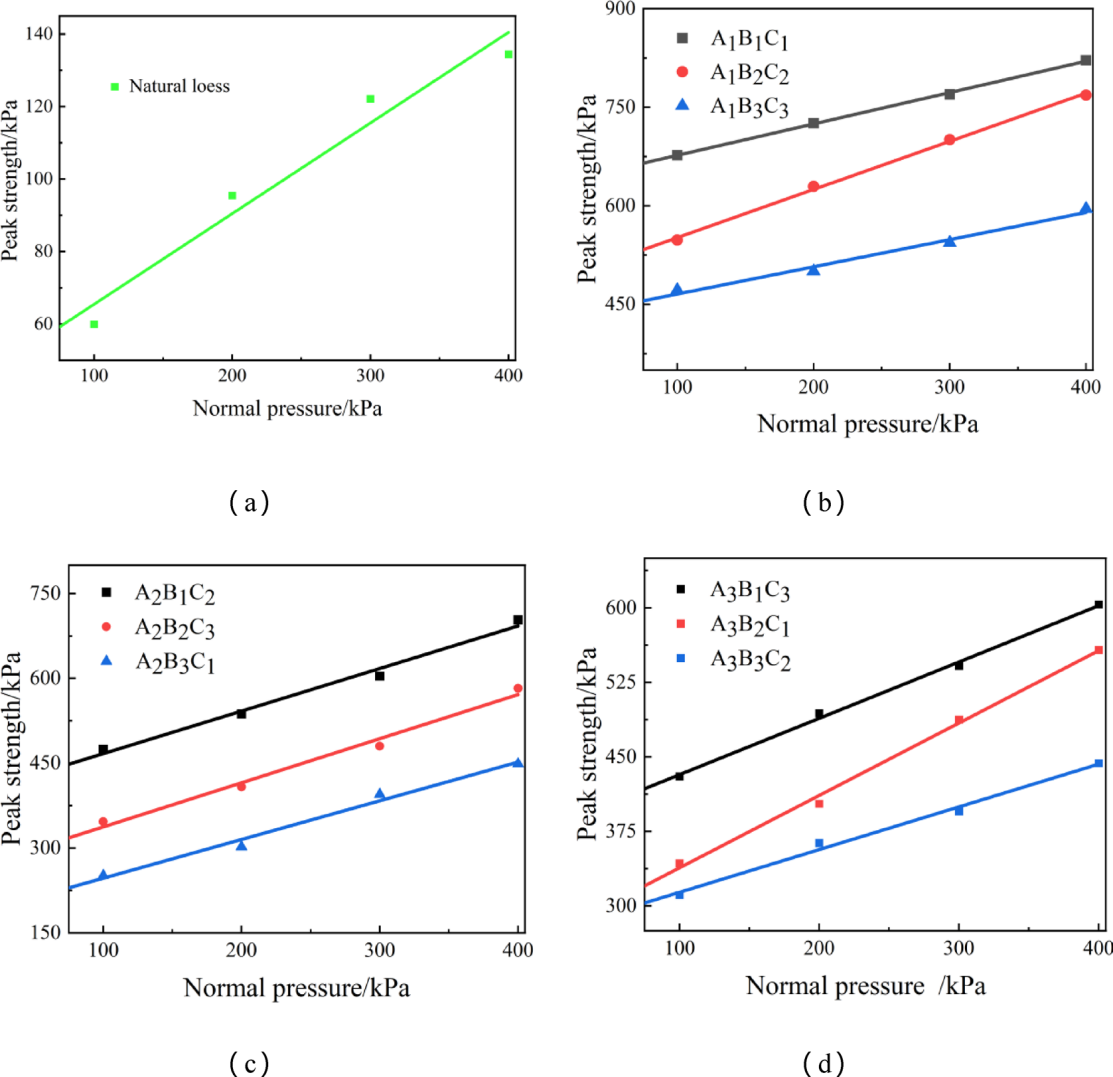
Shear strength variation.

Figure 12 presents the shear strength indicators were obtained from the shear strength fitting curves, where the y-intercept corresponds to cohesion, and the slope represents the internal friction angle^44–46^. As shown in the graph, experimental group A_1_B_1_C_1_ has the highest cohesion, while A_2_B_3_C_1_ has the lowest. The cohesion values increase by 14.6 times and 2.7 times, respectively, compared to pure loess. This is because, in group A_1_B_1_C_1_, the S/B ratio is 2.0. The higher the amount of high-water quick-setting material added to the loess, the more cementitious substances are formed within the soil. These substances create interlocking structures between adjacent soil particles, enhancing the interaction forces and, ultimately, the shear strength^44^.

#### Parametric analysis

##### Effect on cohesion

Based on Fig. 12, by controlling the W/B ratio and analyzing the S/B ratio as a single factor, the following observations were made: loess with a S/B ratio of 2.0 has significantly higher cohesion than when the S/B ratio is 3.0 or 6.0. Additionally, cohesion decreases as the S/B ratio increases^47^. In other words, adding more high-water quick-setting material increases the cohesion. This is because the shear strength of the soil is composed of frictional strength and cohesive strength, with cohesion playing the largest role in shear strength due to the cementitious material’s influence. The addition of high-water quick-setting material enhances cohesion by forming a dense, stable structure within the soil, which strengthens the shear resistance. When analyzing the cohesion at different W/B ratios for the same S/B ratio, the cohesion value is significantly higher at a W/B ratio of 1.0. As the W/B ratio increases, moisture content within the soil also increases, leading to more water between soil particles and in the pores. This reduces cohesion, making the soil more susceptible to shear failure under lateral shear forces.

##### Effect on the internal friction angle

The internal friction angle is a critical parameter for soil shear strength. It is influenced by factors such as the soil’s initial porosity, particle shape, particle gradation, and surface roughness^48^. The internal friction angle for each experimental group was calculated using the slope of the shear strength fitting curves and Coulomb’s formula. The results are as follows: At a W/B ratio of 1.0, the maximum internal friction angle for A_1_B_2_C_2_ is 41.98°, while the angles for A_1_B_1_C_1_ and A_1_B_3_C_3_ are 27.36° and 23.72°, respectively. At a W/B ratio of 1.5, the maximum internal friction angle for A_2_B_2_C_3_ is 44.68°, with angles for A_2_B_1_C_2_ and A_2_B_3_C_1_ of 43.11° and 39.22°, respectively. At a W/B ratio of 2.0, the maximum internal friction angle for A_3_B_2_C_1_ is 41.74°, while A_3_B_1_C_3_ and A_3_B_3_C_2_ have angles of 32.48° and 24.63°, respectively. From these results, it is evident that, for a fixed W/B ratio, a S/B ratio of 3.0 produces the most significant increase in the internal friction angle of loess, followed by a S/B ratio of 2.0. For a fixed S/B ratio, a W/B ratio of 1.5 results in the best improvement in the internal friction angle of loess.

### Effect of high-water quick-setting material on the microstructure of loess

#### SEM analysis

**Fig. 13:**
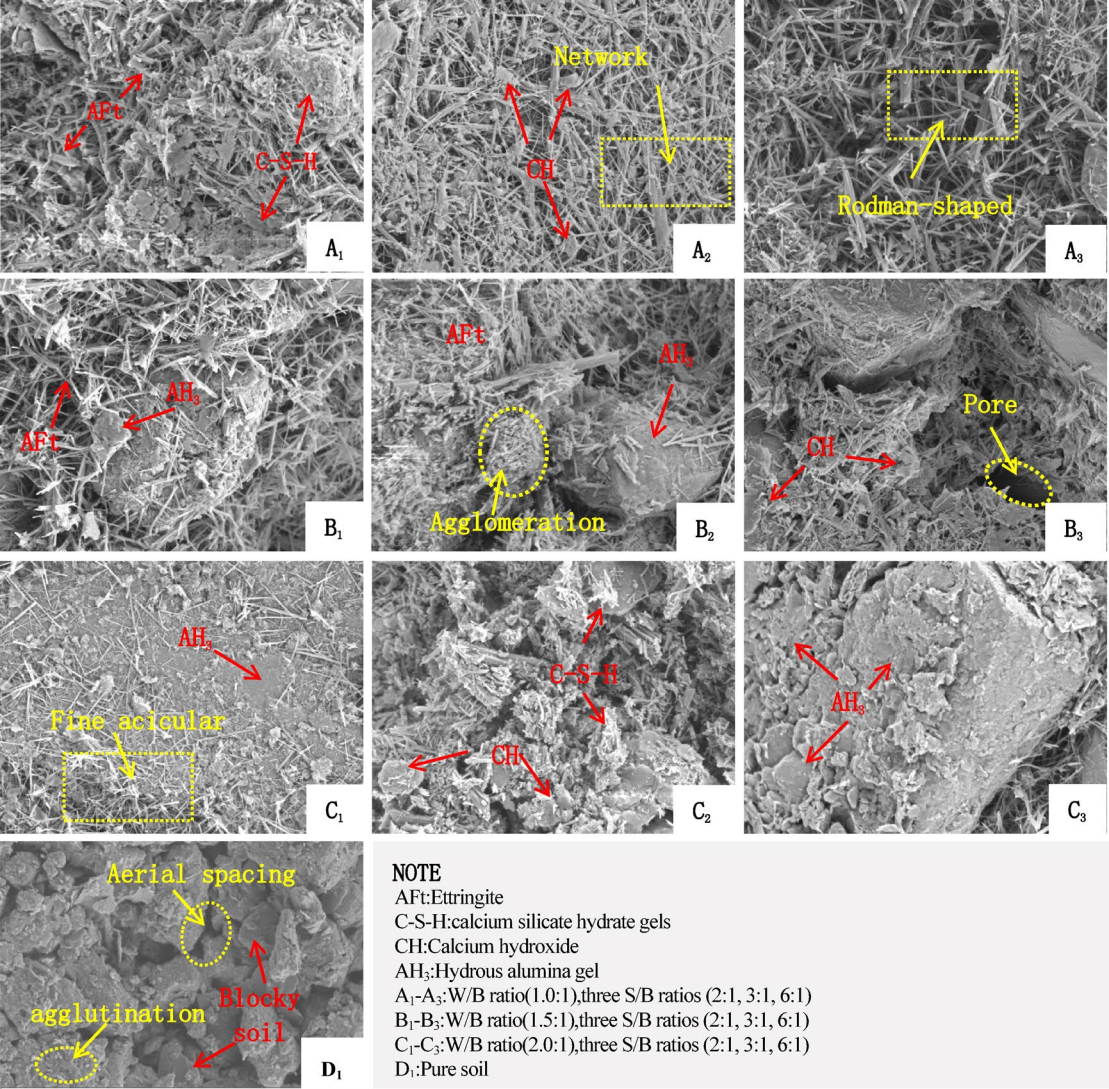
SEM images.

The microstructure of a material directly influences its macroscopic properties^49,50^. The macroscopic performance of materials is fundamentally governed by their microstructure. As shown in Fig. 13D_1_ (2000× magnification), unmodified loess exhibits heterogeneous interparticle pores and distinct structural units. The soil matrix comprises granular particles and aggregates, where silt- to sand-sized particles form a skeletal framework, with finer clay-sized particles acting as cementing agents between larger grains.

These aggregates demonstrate two primary structural configurations: Type I: Coarse silt particles form a rigid skeleton, with angular to subangular fragments (blocky or platy morphology) interconnected by clay minerals and detrital fines. Type II: Clay-dominated agglomerates lacking distinct granularity, consolidated by soluble salts, carbonates, or amorphous oxides. Three pore types dominate the microstructure: (a) skeletal pores (formed between coarse framework particles), the interlocked pores (within densely packed fine aggregates) and the cementation pores (at clay-mineral bonding interfaces). This hierarchical pore-grain architecture directly influences mechanical and hydraulic properties, with skeletal pores governing permeability and cementation interfaces dictating cohesive strength.

As shown in Fig. 13 (A_1_–C_3_), 6000× magnification micrographs of high-water quick-setting material-modified loess reveal abundant acicular, columnar, and amorphous cementitious phases. The mechanical enhancement arises from chemical reactions among CaO, SiO₂, and Al_2_O₃ in the binder system, as detailed below:

1 $${\mathrm{C}}_{{\mathrm{4}}} {\mathrm{A}}_{{\mathrm{3}}} {\bar{\mathrm{S}}} + {\mathrm{18H}} \to {\mathrm{AFm}} + {\mathrm{2AH}}_{{\mathrm{3}}}$$

2 $${\mathrm{3C}}_{{\mathrm{4}}} {\mathrm{A}}_{{\mathrm{3}}} {\bar{\mathrm{S}}} + {\mathrm{2CSH}}_{{\mathrm{2}}} + {\mathrm{34H}} \to {\mathrm{3AFt}} + {\mathrm{4AH}}_{{\mathrm{3}}}$$

3 $${\mathrm{2CS}} + {\mathrm{2H}} \to {\mathrm{C}} - {\mathrm{S}} - {\mathrm{H}} + {\mathrm{CH}}$$

4 $${\mathrm{C}}_{{\mathrm{4}}} {\mathrm{A}}_{{\mathrm{3}}} {\bar{\mathrm{S}}} + {\mathrm{8C}}\bar{\mathrm{S}}\mathrm{H}_{{\mathrm{2}}} + {\mathrm{6CH}} + {\mathrm{74H}} \to {\mathrm{3AFt}}$$

These products collectively establish a “needle-gel-plate” hierarchical microstructure, where AFt crystals bridge macropores, C-S-H gels densify mesopores, and AH_3_/CH optimize nanoscale interfaces^51,52^.

**Fig. 14:**
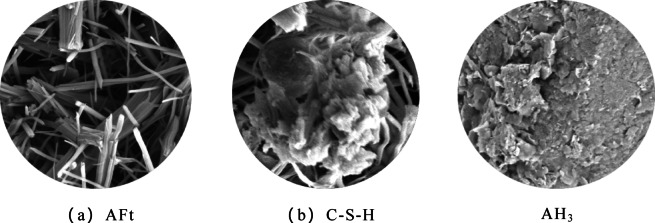
Key hydration products. (**a**) AFt, (**b**) C-S-H.

As illustrated in Fig. 14, the mechanical improvement of loess by the high-water quick-setting material is attributed to the synergistic effects of three key hydration products: (a) Ettringite (AFt), which forms an early-strength skeletal framework through its acicular and columnar crystal morphology. Its expansive properties effectively fill macropores and mitigate shrinkage; (b) the calcium silicate hydrate (C-S-H) gel which enhances long-term strength via nm-level cementation, concurrently improving impermeability and toughness; and the aluminum hydroxide (AH_3_) gel, the main effect of which is to facilitates supplementary cementation and provides aluminum sources for AFt formation. Unlike cement, the hydration products of high-water quick-setting materials can form a three-dimensional network structure with ettringite as the framework and other gels as supplementary components, even under high W/B ratio (W/B > 1.0). This structure effectively encapsulates and immobilizes large amounts of free water, resulting in a solidified body that exhibits both high-water quick-setting and considerable strength.

The W/B ratio critically governs hydration kinetics, ettringite (AFt) crystal growth, and the porosity/density of the cementation network, directly influencing macroscopic strength^53^. SEM analysis revealed that increasing the W/B ratio accelerated hydration rates but reduced AFt abundance while promoting fragile aluminum hydroxide (AH_3_) gel formation. As shown in Fig. 13A_3_–B_1_, AFt crystals transitioned from robust, elongated columns (low W/B ratios) to slender, fragmented needles (high ratios), accompanied by increased porosity and water retention. This microstructural coarsening explains the decline in compressive strength at elevated W/B ratios.

As illustrated in Fig. 13C_1_–C_3_, lower S/B ratios (e.g., 2.0:1) significantly enhanced AFt proliferation and spatial dominance. Specimens like A_1_B_1_C_1_ exhibited densely interlocked AFt networks-rregular columns and needles bridging soil particles and filling pores (Fig. 13b–d). The crystal entanglement restricted particle displacement, while the AFt growth reduced void volume, enhancing compactness. During this period, free water was rapidly converted to crystalline water within AFt or trapped as non-bound water in gel pores, enabling rapid setting.the high early strength of the material stems from this AFt-driven framework, where capillary forces stabilize non-bound water within the nanostructured matrix. This water acts as a “binding-plasticizing” agent, maintaining material stability without compromising rigidity^54–57^.

#### NMR analysis

##### Principles and methodology

Nuclear Magnetic Resonance (NMR) is an analytical technique based on the magnetic properties of atomic nuclei, with hydrogen nuclei (^1^H) being the most commonly studied due to their high abundance, sensitivity, and structural relevance^58^. The principle relies on the precession of hydrogen nuclei in an external magnetic field, generating detectable signals. In soil systems, hydrogen nuclei primarily reside in pore water, where the transverse relaxation time (T₂) correlates with pore size, shape, and connectivity. Thus, T₂ distribution analysis enables inference of soil pore structure^59^.

**Fig. 15:**
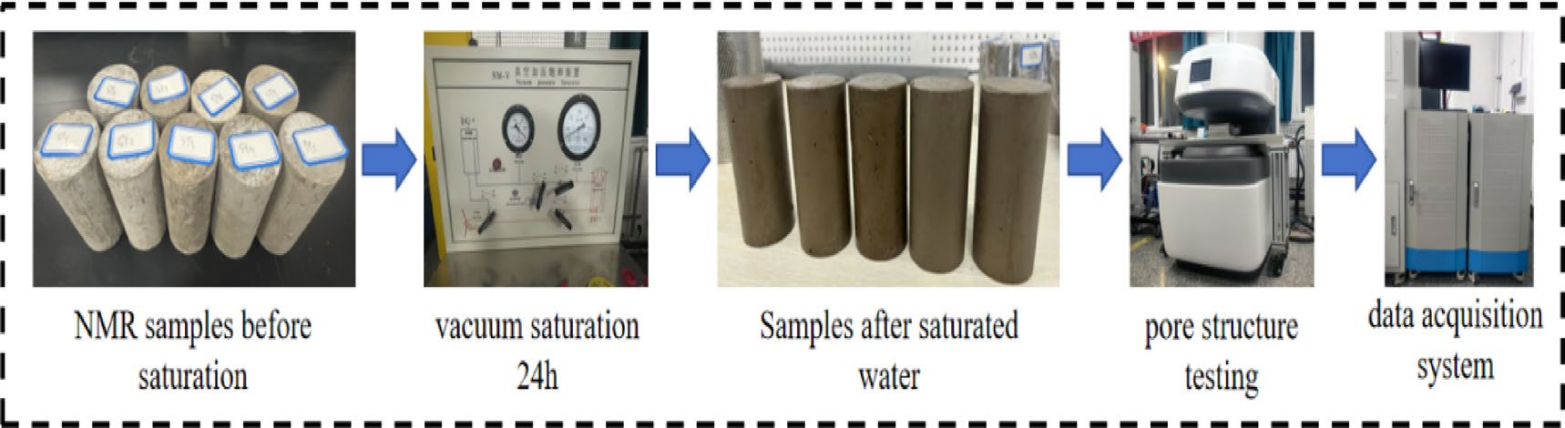
NMR analysis procedure.

In this study, ^1^H NMR was employed to characterize the relaxation behavior of pore water in high-water quick-setting material-modified loess, focusing on pore structure distribution and quantification. Specimens identical in mix proportions and dimensions to those used in unconfined compressive strength tests were water-saturated for 24 h prior to NMR measurements (experimental setup illustrated in Fig. 15). The specimens were vacuum-saturated to evacuate air from pores, followed by water infusion under sustained vacuum to ensure maximal pore-filling. This protocol guarantees that the T₂ spectrum accurately reflects the full pore structure. The relationship between relaxation time and pore structure for modified loess is expressed as:

5 $$\frac{1}{{T}_{2}}={\rho}_{2}\times\frac{S}{V}={\rho}_{2}\times\frac{{F}_{S}}{R}={\rho}_{2}\times\frac{3}{R}$$

##### T₂ Spectrum distribution

**Fig. 16:**
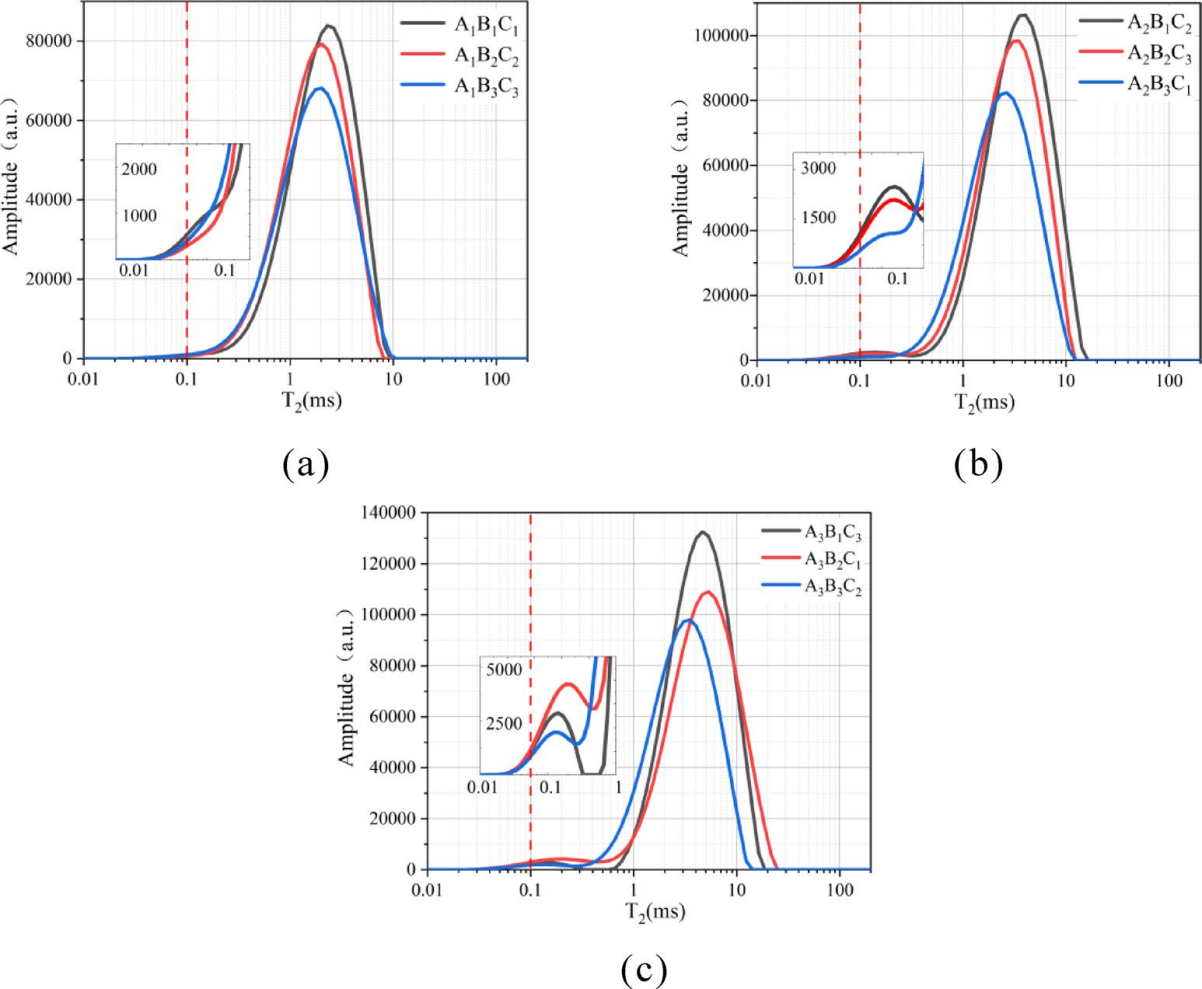
T_2_ relaxation time distribution curves.

Analysis of Fig. 16 reveals distinct primary peaks in the T₂ distribution curves for all nine experimental groups, with high amplitudes concentrated in the 1–10 ms range. Figure 16a exhibits a single dominant peak, while Fig. 16b and c display bimodal distributions. For the first peak, there is a smaller amplitude (1,977.92–4,226.36) within 0.01–0.43 ms, while the Larger amplitude (68,179.3–132,584.05) spanning 0.43–24.77 ms was found from the second peak. These bimodal features reflect pore connectivity in the soil. The horizontal axis (proportional to pore size) and vertical axis (signal amplitude, representing pore quantity) define the curves, with the total area under the curve corresponding to porosity. Amplifies peak amplitudes and integrated curve areas with the increased W/B ratio. when the S/B ratio (at fixed W/B ratios) was increased, the peak amplitudes, integrated areas, and maximum curve values experienced the decrease.

##### T₂ Spectrum distribution

**Fig. 17:**
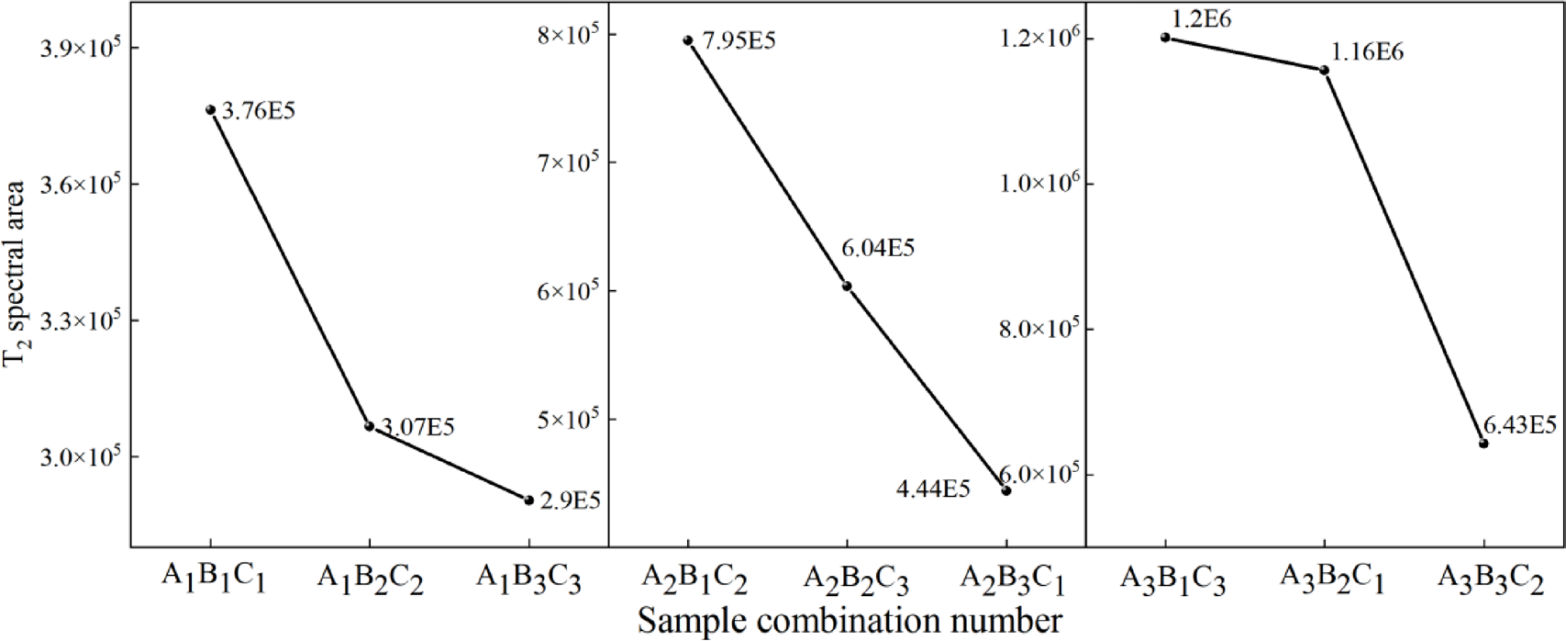
T_2_ spectrum area variation curves.

According to nuclear magnetic resonance (NMR) principles, the total area under each inverted T₂ curve represents the relative water content of the specimen, allowing the determination of pore volume in porous media through water volume calculations. The peak areas in Fig. 15 were analyzed using Origin software, as illustrated in Fig. 17. The T₂ spectrum areas under different mix ratios followed the descending order: W/B ratio (2:1 > 1.5:1 > 1:1) and S/B ratio (2:1 > 3:1 > 6:1). This indicates that higher W/B ratios increase pore quantity and porosity in loess, while higher S/B ratios reduce porosity. Specimen A_1_B_3_C_3_ exhibited the smallest area (minimum porosity) among the nine groups, whereas A_3_B_1_C_3_ showed the largest area, which was 4.14 times greater than the minimum. At fixed W/B ratios, increasing the S/B ratio resulted in area reductions as follows: 1:1 (19% and 28%), 1.5:1 (24% and 26%), and 2:1 (4% and 46%). Conversely, at fixed S/B ratios, increasing the W/B ratio led to area increments: 2:1 (69% and 34%), 3:1 (73% and 48%), and 6:1 (55% and 31%).

##### T₂ pore structure evolution

**Fig. 18:**
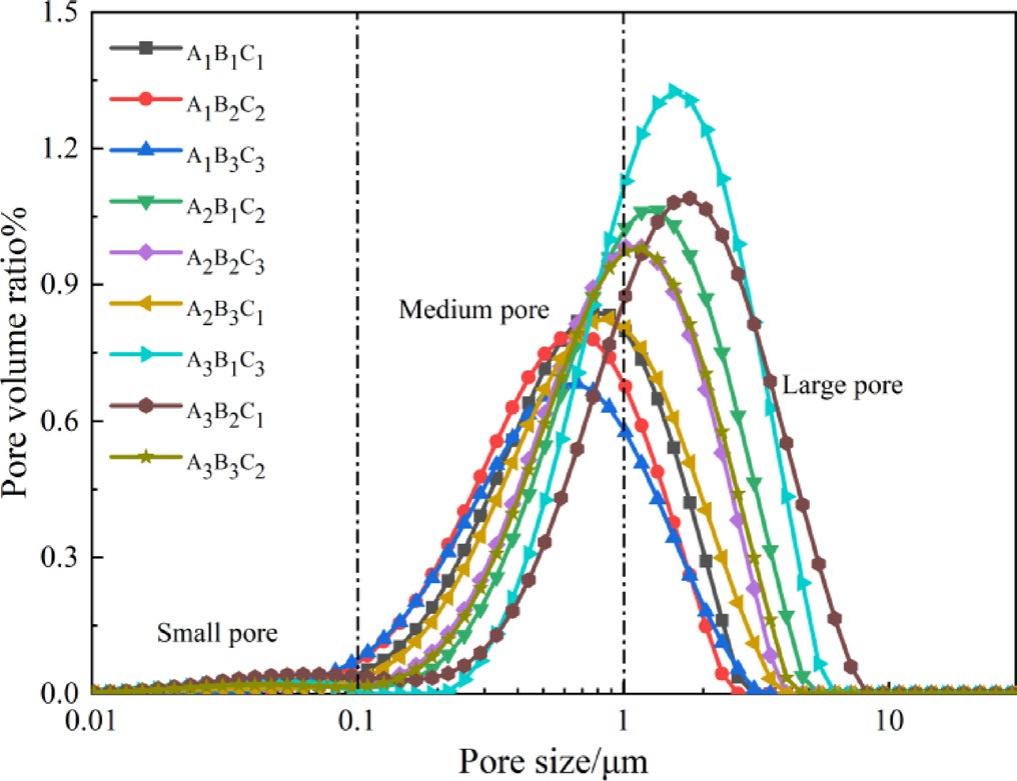
Pore distribution curves of soil samples.

Currently, there is no unified standard for pore size classification in high-water quick-setting material-modified loess. This study adopts the classification method proposed by Cheng et al.^60–62^, dividing pores into three categories via NMR-detected hydrogen atoms: micropores (< 0.1 μm), mesopores (0.1–1 μm), and macropores (> 1 μm). As shown in Fig. 18, mesopores dominate (W/B ratio = 1:1), followed by macropores and micropores. Increasing the W/B ratio shifts the peak rightward, amplifying macropore dominance. Conversely, at fixed W/B ratios, increasing the S/B ratio shifts peaks leftward, reducing peak amplitude, pore size, and quantity.This behavior stems from microstructural changes: hydration-generated ettringite transitions from thick, elongated columns to slender, fragmented needles, increasing pore quantity and structural looseness. Higher S/B ratios reduce pore size and quantity due to diminished ettringite formation, where loess particles dominate the matrix. These trends align with SEM observations, confirming that densely interlocked ettringite crystals (at lower S/B ratios) refine pore networks, whereas particle-dominated systems (higher ratios) coarsen them.

## Engineering applications and durability assessment

### Early-stage strength analysis

**Fig. 19:**
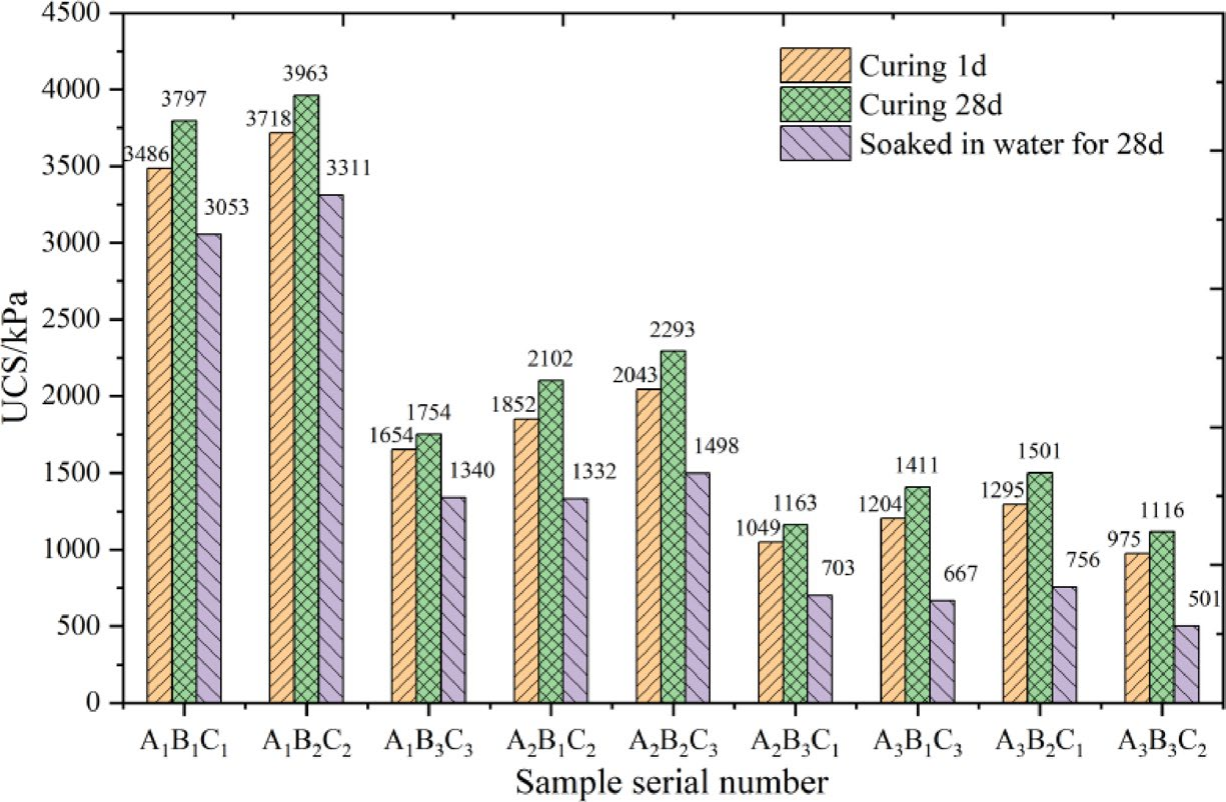
Comparison in UCS for different samples.

Figure 19 illustrates the strength enhancement of loess modified with high-water quick-setting material and its evolution over curing periods (1-day and 28-day unconfined compressive strength, UCS). The modified loess exhibits rapid setting and high early strength, governed predominantly by the W/B ratio. Specimens with a low W/B ratio (1:1) achieved the highest 1-day UCS (3486.3–3717.5 kPa) but showed limited 28-day strength growth (6–9%). Conversely, high W/B ratio specimens (2.0:1) demonstrated substantial late-stage strength growth (14.5–17.2%) but the lowest absolute strength (1116.1–1501.3 kPa), reflecting a “high growth rate–low base” characteristic. Reducing the S/B ratio improved initial strength but suppressed long-term growth (e.g., S/B ratio 6:1 specimens exhibited > 50% higher growth rates than 2:1 specimens).

### Water immersion test results

To evaluate the water stability of modified loess, in accordance with the strength test under saturation state specified in Standard for soil test methods (GB/T 50123 − 2019), this paper tests the Unconfined compressive strength of specimens under saturation state using the Vacuum saturation method. The strength change of the material is evaluated by calculating the Water immersion softening coefficient K (the ratio of Unconfined compressive strength Before water saturation to Unconfined compressive strength after 28 days of water saturation). A larger K value indicates better water stability of the specimen. The results are shown in Fig. 19; Table 6. Specimen A_1_B_2_C_2_ (W/B ratio 1:1 + S/B ratio 3:1) exhibited optimal performance (K = 0.89), whereas A_3_B_3_C_2_ (2.0:1 + 6:1) showed the lowest K (0.51) due to excessive porosity-induced cementation collapse. Overall, water resistance correlated positively with cementation density, with low W/B ratios (≤ 1:1) achieving K > 0.8. Mechanistically, low W/B ratios promote rapid ettringite (AFt) formation, creating dense matrices, while high ratios prioritize C-S-H gel development, increasing porosity despite late-stage strength gains. Moderate W/B ratios (1.5:1) balance hydration and porosity, whereas excessive ratios (2.0:1) degrade both strength and water resistance.

**Table 6:** Durability assessment parameters.

Number	28-day strength growth rate (W)	Water immersion softening coefficient (K)
A_1_B_1_C_1_	8.9%	0.88
A_1_B_2_C_2_	6.6%	0.89
A_1_B_3_C_3_	6.0%	0.81
A_2_B_1_C_2_	13.5%	0.72
A_2_B_2_C_3_	12.2%	0.73
A_2_B_3_C_1_	10.8%	0.67
A_3_B_1_C_3_	17.2%	0.55
A_3_B_2_C_1_	15.9%	0.58
A_3_B_3_C_2_	14.5%	0.51

### Engineering applications

In engineering practice, optimizing the composite mix ratios of high-water quick-setting materials and collapsible loess is critical for enhancing the mechanical performance of subgrade filling projects. Maintaining the W/B ratio at a low level (W/B ≤ 1.5) significantly improves compressive strength by reducing post-hardening porosity and enhancing compactness. Concurrently, the S/B ratio is recommended to be controlled within 2.0–3.0. Excessively high S/B ratio may lead to insufficient cementation, compromising bonding performance, while overly low S/B ratio without proportional strength gains. For subgrade filling, a mix ratio of W/B ratio = 1.5 and S/B ratio = 3.0 balances strength requirements with material usage, avoiding excessive binder consumption. This ratio serves as a practical reference for similar projects. Furthermore, an engineering adaptation scheme is proposed by evaluating the improvement effects of the mechanical properties of loess under different W/B and S/B ratios, the water resistance results in water stability tests, and comparing with the strength requirements in actual engineering, as shown in Table 7. It is noteworthy that all proposed mix designs meet the unconfined compressive strength requirements (typically ranging from 1.5 to 3.0 MPa) specified in the Technical Specifications for Construction of Highway Roadbase (JTG/T F20-2015) for cement-stabilized soils used in base and subbase courses of various highway classes. The rapid construction mix achieves high early strength, making it suitable for emergency repair scenarios. The long-term structural mix provides superior sustained strength and durability, rendering it appropriate for permanent subgrade construction.

**Table 7:** Engineering adaptation schemes.

Engineering requirement	Recommended mix ratio	Performance	Application scenario
Rapid construction	W/B ratio 1:1 + S/B ratio 2:1	1-day strength > 3400 kPa, K > 0.85	Emergency subgrade repair
Long-term stable structure	W/B ratio 1:1 + S/B ratio 3:1	28-day strength > 3900 kPa, K = 0.89	Permanent subgrade construction
Low-cost temporary works	W/B ratio 1.5:1 + S/B ratio 3:1	28-day strength ≈ 2300 kPa, requires waterproofing (K = 0.73 )	Temporary roads, earth-filling projects

## Conclusions

This study proposes a novel approach for improving collapsible loess using high-water quick-setting materials, with experimental and theoretical investigations validating its practicality and effectiveness. The findings provide a theoretical foundation for promoting this material in road engineering. Key conclusions are as follows:

1. Unconfined compressive strength (UCS) tests demonstrated that high-water quick-setting materials significantly enhance loess compressive strength. Orthogonal experimental sensitivity analysis revealed the following order of influence on UCS: W/B ratio > S/B ratio > loading rate.
2. Direct shear tests indicated that modified specimens exhibited markedly higher shear strength than untreated loess. Cohesion peaked at W/B ratio = 1.0 and S/B ratio = 2.0, while the internal friction angle reached its maximum at S/B ratio = 3.0 and W/B ratio = 1.5.
3. SEM analysis identified hydration products, including ettringite (AFt) and calcium silicate hydrate (C-S-H) gel, which interlock with loess particles to form a 3D network. This microstructure enhances both compressive and shear strength.
4. NMR results revealed that higher W/B ratios increase pore quantity and porosity, while higher S/B ratios reduce porosity. Increasing W/B ratio amplified macropore dominance, whereas increasing S/B ratio induced a leftward peak shift, reducing pore size and quantity.
5. The optimal mix ratio for high-water quick-setting material-modified loess was determined as W/B ratio ≤ 1.5 and S/B ratio = 2.0–3.0. This formulation ensures complete encapsulation of soil particles by cementitious materials and formation of a stable spatial network structure. It effectively compensates for the cementation deficiency in low-activity soils, significantly enhancing mechanical properties while meeting the critical strength and durability requirements for road base materials.
6. This study conducted laboratory tests on the mechanical properties of collapsible loess treated with high-water quick-setting material. The variations in mechanical parameters of the loess under different W/B ratios and S/B ratios were obtained. Further investigation into the field application of this material in road construction is essential to validate its performance in reinforcing collapsible loess.

This study presents promising results, yet future work should address the environmental impact and management of SO₃ emissions from production. Optimizing mix proportions, production methods, and construction techniques remains crucial for selecting effective stabilizers. high-water quick-setting materials provide an efficient and eco-friendly potential alternative to cement for stabilizing collapsible loess.

## Data Availability

The datasets used and/or analysed during the current study available from the corresponding author on reasonable request.
